# Hormonal Implications of SARS-CoV-2: A Review of Endocrine Disruptions

**DOI:** 10.1155/sci5/7305185

**Published:** 2025-01-12

**Authors:** Aliya Yskak, Yevgeniy Sokharev, Kuanysh Zhumalynov, Elizaveta Koneva, Natalia Afanasyeva, Dmitri Borodulin, Dmitrii Babaskin, Almabek Nugmanov, Murat Nurushev, Vadim Chashkov

**Affiliations:** ^1^Research Institute of Applied Biotechnology, Akhmet Baitursynuly Kostanay Regional University, Kostanay, Kazakhstan; ^2^Faculty of Soil Science, Lomonosov Moscow State University, Moscow, Russia; ^3^Pathological Anatomy Department, Municipal State Company “Kostanay Regional Pathoanatomical Bureau” of the Health Department of the Akimat of the Kostanay Region, Kostanay, Kazakhstan; ^4^Department of Natural Sciences, Akhmet Baitursynuly Kostanay Regional University, Kostanay, Kazakhstan; ^5^Department of Sports Medicine and Medical Rehabilitation, Sechenov University, Moscow, Russia; ^6^Resource Center “Medical Sechenov Pre-University”, Sechenov University, Moscow, Russia; ^7^Department of Technology of Storage and Processing of Fruits, Vegetables and Plant Growing Products, Russian State Agrarian University-Moscow Timiryazev Agricultural Academy, Moscow, Russia; ^8^Department of Pharmacy, Sechenov University, Moscow, Russia; ^9^Higher School of Natural Sciences, Astana International University, Astana, Kazakhstan

**Keywords:** coronavirus infection, endocrine system, functional activity, atividade funcional, infeção por coronavírus, sistema endócrino

## Abstract

To improve medical care and rehabilitation algorithms for patients affected by severe acute respiratory syndrome coronavirus 2 (SARS-CoV-2), it is important to evaluate and summarize the available data on the effect of coronavirus infection (COVID-19) on the endocrine system. The purpose of this review was to study the effect of COVID-19 on the endocrine system. The scientific novelty of this study is the evaluation of the effect of coronavirus infection on the endocrine system and the potential effect of hormones on susceptibility to COVID-19. The results of this review show that the endocrine system is vulnerable to disorders caused by COVID-19, mainly thyroid dysfunction and hyperglycemia. The information in the published literature mentioned here contains some unclear aspects and contradictory data, but much remains to be studied and clarified regarding the impact of COVID-19 on the endocrine system. In particular, this concerns the study of the hyperglycemic status of patients who have had coronavirus infection, which is extremely important for the future metabolic health of COVID-19 survivors. This review contributes to the scientific discourse by systematically synthesizing disparate studies to identify patterns, gaps, and emerging trends in the literature concerning the effects of COVID-19 on the endocrine system. By integrating these findings, this study offers a novel perspective on potential hormonal interactions influencing COVID-19 susceptibility and outcomes, proposing new hypotheses and frameworks for future research.

## 1. Introduction

Human coronavirus (Coronaviridae, Coronavirus) [1, 2] was first isolated by D. Tyrrell and M. Bynoe in 1965 from patients with acute respiratory viral disease. In 1967, K. McIntosh isolated coronavirus strains in tracheal cell culture. Previously, coronavirus infection was registered throughout the year. An increase in morbidity was noted in winter and early spring, when its epidemic significance ranged from 15% to 33.7% [3]. Children get sick 5–7 times more often than adults. The infection is spread by airborne transmission and via fecal–oral and contact routes. Patients with a clinically pronounced or suppressed form of the disease are the source of infection [4, 5]. Among hospitalized patients, 12.4% (with fluctuations in some years equaling 6.8%–28.6%) had acute respiratory viral infection (ARVI) [6, 7]. Coronaviruses, as a rule, lead among other viruses in the etiology of nosocomial infections. Immunity after the disease is short-lived and does not protect against reinfection. In November 2002, in the Chinese Guangdong Province, a previously unknown disease was first identified and described. This illness, which later became known as severe acute respiratory syndrome (SARS), is characterized by symptoms that do not respond to conventional antibiotic treatments. Its emergence is particularly alarming due to the rapid spread of infections, indicating that it is a highly contagious virus. SARS is characterized by severe respiratory symptoms, fever, and, in more serious cases, acute respiratory distress, which requires mechanical ventilation [8].

In the spring of 2003, the etiological agent responsible for the outbreak, known as SARS-CoV, which belongs to the Coronaviridae family, was discovered. This virus, confirmed to be of animal origin, was isolated due to its distinctive pattern of transmission and clusters of cases, which strongly suggested a viral origin [9].

On December 11, 2020, the World Health Organization (WHO) gave the official name of COVID-19 to the infection caused by the 2019-nCoV coronavirus. On February 11, 2020, the International Committee on the Taxonomy of Viruses officially named the virus SARS coronavirus 2 (SARS-CoV-2). The designation was selected due to the virus's genetic similarity to the pathogen responsible for the 2003 SARS outbreak. While distinct, the two viruses share a genetic connection. On March 11, 2020, the spread of the virus was recognized as a pandemic [10].

SARS-CoV-2 is a single-stranded RNA-containing virus with a corona-like S glycoprotein. The full-genome sequence of the SARS-CoV-2 virus is 96% similar to that of the SARS-like coronavirus of bats [11]. This virus is 79.6% identical to SARS-CoV-1; some encoded proteins, such as the main proteinase of coronavirus, papain-like proteinase, and RNA-dependent RNA polymerase [12], have 96% similarity to SARS-CoV [12].

To penetrate the host cell and ensure the fusion of the virus membrane with the host cell membrane during infection, SARS-CoV-2 uses a surface spike glycoprotein (S). S glycoprotein is a trimeric protein. It plays a key role in ensuring the survival of coronaviruses because it not only acts as an important functional part of the virion but also completely ensures attachment and fusion with the membranes of the host cell. In addition, the S protein, which is the largest surface protein of coronaviruses, determines the solubility of viral particles and, as a consequence, the contagiousness of SARS-CoV-2 [13]. SARS-CoV-2 exhibits a high degree of homology to SARS-CoV [14]. It penetrates the host cell through the interaction between the S protein of the virus and the human angiotensin-converting enzyme 2 (ACE2) receptor. However, the molecular mechanism of this connection, as well as the evolution of SARS-CoV-2, remains poorly understood. Nevertheless, when comparing the complexes that form the analyzed viruses with ACE2, SARS-CoV-2 binds to the enzyme with a greater affinity.

The main target cells of the coronavirus are type II pneumocytes and enterocytes. The interaction of the SARS-CoV-2 S protein with the ACE2 receptor initiates a conformational change in the S protein, facilitating its proteolytic processing by the host cell protease TMPRSS2. Moreover, Lambert et al. [15] demonstrated that ACE2 itself is subject to metalloproteinase-mediated ectodomain shedding, which is predominantly mediated by ADAM17. This regulated shedding, influenced by various stimuli, underscores the complex regulatory mechanisms influencing ACE2 activity and availability and is pivotal for understanding viral entry and host responses. Thus, a nucleocapsid enters the cell. After the release of chromosomal RNA, all cell resources are redirected to the synthesis and assembly of new virions, which eventually begin to bud from the cell membrane, destroying it and infecting neighboring cells, and this process is repeated many times [16].

The human carboxypeptidase ACE2 is encoded by the ACE2 gene located on chromosome 22 [17]. ACE2 is a type I transmembrane protein with an extracellular N-glycosylated N-terminal site containing a carboxypeptidase site, as well as a short intracellular C-terminal cytoplasmic tail [18]. The N-terminal peptidase domain is the site at which ACE2 associates with SARS-CoV. There are also two forms of the ACE2 protein: cellular (membrane-bound) and circulating (soluble). The cellular form is a full-fledged protein synthesized in large quantities by pneumocytes or enterocytes of the small intestine.

ACE and ACE2 are indispensable components of the renin–angiotensin system (RAS), whose tasks are to maintain homeostasis of the cardiovascular system and the functioning of various organs and to regulate systolic pressure and osmotic and electrolyte balance. It is necessary to note the common properties and differences in these 2 enzymes. Both enzymes are involved in the conversion of angiotensin I (AngI). ACE catalyzes its conversion to angiotensin II, and ACE2, which binds to the latter, converts it to angiotensins III, IV, and V [19]. An excess of these angiotensins can cause the development of respiratory distress syndrome (RDS) in adults because, with RDS, the secretion of ACE2 increases [20].

In addition, the mechanism underlying the effect of bradykinin on chronic cough is known, and among other effects, it appears in people taking various ACE inhibitors (more than 4%). These medicines, as well as sartans, ultimately lead to hypersecretion of ACE2. Although ACE inhibitors act directly, increasing the concentrations of bradykinin and, accordingly, ACE2, sartans do so indirectly. By blocking angiotensin receptors, they increase the concentration of angiotensins I and II in the blood, which, if the alternative transition is disrupted, can reflexively increase the production of ACE2, especially with their chronic use [21].

Angiotensinogen is synthesized in the liver, after which it is converted by renin into AngI and then, with the participation of ACE, into AngII. AngII is a key component of the RAS and binds to the type I angiotensin receptor (AT1R). This interaction leads to the reduction in bronchial smooth muscles, proliferation of fibroblasts in the lungs, apoptosis of alveolar epithelial cells, increased vascular permeability in lung tissue, and acute RDS [22].

ACE2 acts as a counterregulator of the activity of the ACE–AngII–AT1R complex and hydrolyzes AngII to Angl-7 [23]. As a consequence, the metabolic pathway of angiotensin is not inhibited. This situation only aggravates the infection process and the development of inflammation, and the cytokine storm disrupts the function of not only the respiratory tract but also the cardiovascular and other organ systems [24].

The concentration of ACE2 may increase after ischemic stroke. This compensatory reaction is aimed at eliminating excess Ang1-7 and providing protective effects by balancing AngII.

ACE2 is involved in the pathological processes of renal tissue, although the exact mechanism has not yet been fully established. Mice with ACE2 deficiency develop glomerulosclerosis and albuminuria [25]. A decrease in the concentration of ACE2 causes an imbalance of AngII, which is involved in renal inflammation and fibrosis, explaining, at least in part, progressive kidney damage.

An important nonpeptidase function of ACE2 is its participation in the transport of amino acids through the wall of the small intestine. One of these amino acids is tryptophan, which regulates the secretion of antimicrobial peptides that affect the composition of the intestinal microbiome. This explains the presence of colitis in mice with ACE2 deficiency, in which tryptophan transport is disrupted and tryptophan deficiency leads to dysbiosis and inflammation.

Therefore, for people with chronic diseases such as hypertension, coronary heart disease, and diabetes mellitus, infection with SARS-CoV-2 is extremely dangerous. In these diseases, the metabolic pathway of angiotensin is excessive, and the acquisition of a coronavirus infection with a high level of virulence and mutagenicity seriously aggravates the course of concomitant diseases and, more likely, can lead to severe conditions and even death.

When studying the material of postmortem studies, the most important factor to consider is the factor of autolytic changes.

Autolysis is the process of biological organisms evolving to decompose their structures at different levels by hydrolytic means.

The study of the features of the autolysis process in various biological objects and at different levels of the organization of the biological system makes it possible to identify some general principles that characterize it as a phenomenon in general and distinguish it from other phenomena.

1. The principle of evolutionism is autolysis, which is an evolutionarily fixed process.
2. The principle of enzymatic hydrolytic decomposition means that autolysis is carried out by hydrolases of the biological object itself.
3. The principle of autodecomposition (self-digestion) means that the process is always directed at the structure of a biological object.
4. The principle of multicomponent decomposition means that substances of different classes are split during autolysis.
5. The principle of heterochrony means that different biological structures undergo autolysis at different times.

The morphological manifestations of autolysis are different at different levels of biological organization.

The intensity of autolytic processes varies significantly across different tissue types, reflecting a complex interplay of enzymatic activities and tissue-specific properties. Epithelial and blood tissues, characterized by their rapid turnover rates and high enzymatic activity, are particularly prone to rapid autolysis postmortem. In contrast, connective, cartilaginous, and bone tissues exhibit greater resistance, likely due to their denser matrix and lower metabolic rates. Muscle tissue, however, occupies an intermediate position, experiencing a moderate rate of autolysis that can influence the interpretation of postmortem findings [26].

Under the same exogenous conditions, differences in the degree of autolysis development are associated with the peculiarities of the structure and function of the tissue (i.e., with endogenous factors).

For example, in striated muscle tissue for a certain period, the progression of autolysis is restrained by postmortem rigor mortis, and autolysis manifests itself fully only after its resolution. There is a relatively low concentration of proteolytic enzymes in nervous tissue; the same seems to apply to connective tissue [27].

Noncellular tissue components greatly influence autolytic reactions. A significant proportion of connective tissue, cartilage, and bone is composed of fibrous structures and intercellular matter, which include structural proteins, glycoproteins, and glycosaminoglycans (mucopolysaccharides) that are resistant to the action of hydrolytic enzymes. Bone tissue contains a large admixture of inorganic substances intact to autolysis (up to 70%) [10, 28].

The intensity of autolytic processes may also vary within the same group of tissues. For example, in most cases, autolytic changes in the epithelium of pancreatic acini occur faster than in liver cells. The epithelium of the renal tubules undergoes autolysis at different rates, which is due to differences in the functional and biochemical orientation of various departments of the nephron.

The progression of autolysis in tissues and organs with high regenerative capabilities underscores a complex biological interplay influenced by intrinsic tissue properties and enzymatic activities. More differentiated tissues, such as epithelial cells, often exhibit rapid autolytic processes due to their high metabolic rates and frequent cellular turnover [29]. In organs with robust regenerative systems, such as the liver, the presence of stem cells and a high capacity for regeneration often lead to a delayed onset of autolysis. These tissues maintain their structural integrity longer, which can be crucial for medical research involving postmortem examinations.

Therefore, SARS-CoV-2 and its strains will likely affect the health systems of countries worldwide. In addition, the effects of COVID-19 extend not only to the respiratory system but also to other tissues and organs. Therefore, it is important to deepen our understanding of violations of the physiological functions of COVID-19.

The first reports of cases of SARS-CoV-2 infection indicated the possibility of a potentially clinically significant effect on the endocrine system.

To date, the risk factors for mortality from COVID-19 are advanced age, male sex, and concomitant chronic diseases, such as hypertension, diabetes mellitus, chronic obstructive pulmonary disease (COPD), renal failure, cardiovascular disease (CVD), and malignant neoplasms [30–32].

To date, a sufficient amount of research has described the effect of COVID-19 on the endocrine function of the human body. This effect extends to a wide range of glands, from the pancreas, thyroid gland, testis, ovaries, and adrenal glands to the pituitary gland and epiphysis [1, 33]. However, the effect of COVID-19 on endocrine function has yet to be fully elucidated.

Thus, it is important to evaluate and summarize the available data on the impact of COVID-19 on the endocrine system to contribute to in-depth studies and to assist in improving the medical care and rehabilitation of patients affected by SARS-CoV-2 in the future.

The primary objective of this review is to synthesize and critically evaluate the literature regarding the impact of COVID-19 on the endocrine system, with a focus on identifying gaps in current knowledge.

The scientific novelty of this review lies in its comprehensive analysis of how COVID-19 impacts various components of the endocrine system, coupled with an examination of how hormonal variations might influence susceptibility to and outcomes of the infection. This review also highlights emerging trends and inconsistencies in the current research, proposing suggestions for future directions.

## 2. Materials and Methods

To study the effect of COVID-19 on the endocrine system, a selection of sources was carried out for the period from 1990 to 2023. The sources were selected mainly from the Scopus and Web of Science databases using the following keywords: coronavirus, COVID-19, SARS-CoV-2, endocrine system, pituitary gland, thyroid gland, pancreas, adrenal glands, testes, and ovaries. The studies included in this review were peer-reviewed articles written in English that specifically addressed the physiological and pathological effects of COVID-19 on the endocrine system. Reviews, original research articles, and meta-analyses were considered for inclusion. As a result, 103 sources were selected for the review.

## 3. Results

### 3.1. SARS-CoV-2 and the Endocrine System

SARS-CoV and SARS-CoV-2 enter target cells by binding their S protein to the ACE2 receptor, followed by the priming of the S protein by the host cell's TMPRSS2 enzyme. In humans, the mRNAs for ACE2 and TMPRSS2 are found in various endocrine tissues, including the hypothalamus, pituitary gland, thyroid, adrenal glands, ovaries, testes, and pancreatic islets [34]. Investigations into post-COVID-19 pathophysiology have revealed substantial SARS-CoV-2 infection of endocrine cells, particularly in the adenohypophysis, while sparing the neurohypophysis. This selective tissue tropism and associated lymphocytic infiltrates suggest an autoimmune component potentially provoked by molecular mimicry [35]. Recent studies suggest that SARS-CoV-2 may also provoke autoimmune reactions in the adrenal glands, a phenomenon that can be partially attributed to molecular mimicry. Notably, the impact of the virus on adrenal function has been observed through its interaction with ACTH receptors, which could lead to adrenal insufficiency and contribute to the severity of COVID-19 symptoms [36].

Central hypocorticism, a major dysfunction of the hypothalamic–pituitary–adrenal (HPA) axis, was observed in 39% of survivors of SARS-CoV-2 infection 3 months after recovery. Significant changes were observed in adenohypophysial endocrine cells of SARS-CoV patients and increased serum levels in LH and prolactin hormones, with a decrease in GH and TSH hormones in SARS-CoV and SARS-CoV-2 patients. Hyponatremia cases, possibly due to hypersecretion of antidiuretic hormone, were reported among patients with COVID-19, and some reports suggest that COVID-19 could pose a risk factor for pituitary infarction, while patients with hypopituitarism, Cushing's syndrome, and adrenal insufficiency may be susceptible to severe COVID-19 [37].

The molecular mimicry strategy as reported by [38] between the SARS-CoV amino acid sequences and host ACTH may contribute to adrenal insufficiency. Recent studies have suggested that molecular mimicry between the SARS-CoV-2 spike protein and autoantigens in endocrine tissues may contribute to the pathogenesis of autoimmune endocrinopathies in COVID-19 patients [39]. For instance, the identification of peptides shared between the virus and thyroid, adrenal, and pancreatic tissues provides a molecular basis for the autoimmune responses observed in these glands, potentially exacerbating conditions such as thyroiditis, Addison's disease, and type 1 diabetes [39].

Moreover, the findings underscore the importance of careful antigen selection in vaccine development to mitigate the risk of vaccine-induced autoimmunity. As demonstrated by previous findings, cross-reactive relationships between viral infections and vaccinations have led to autoimmune diseases such as narcolepsy and Guillain–Barré syndrome. Vojdani, Vojdani, and Kharrazian [40] emphasized the necessity of analyzing vaccine antigens for tissue cross-reactive epitopes to prevent potential adverse effects.

Few cases of hypothyroidism were observed in patients affected by SARS-CoV and SARS-CoV-2, with a prevalence of 5%–6%. In SARS-CoV patients, thyroid abnormalities were noted, including changes in follicular and parafollicular cells as well as reduced levels of serum thyroxine and triiodothyronine. Additionally, cases of subacute thyroiditis and Graves' disease were reported among individuals with COVID-19, although no evidence was found linking subacute thyroiditis to SARS-CoV infection.

Diabetes mellitus, a common concomitant disease, was observed in 10%–22% of COVID-19 patients, and this was associated with increased severity and mortality. mRNA, ACE2, and TMPRSS2 proteins are highly expressed in human pancreatic islets and ductal and acinar cells, and according to a study by [41], type 1 diabetes mellitus (DM1) in children in the United Kingdom first increased during the COVID-19 pandemic, thereby creating a connection between severe COVID-19 and increased blood glucose levels.

COVID-19 has been linked to ketoacidosis, particularly in 77% of patients with preexisting type 2 diabetes mellitus (DM2), as well as pancreatitis and elevated levels of amylase or lipase, indicating a possible impact of SARS-CoV-2 on the exocrine pancreas. Evidence from immunohistochemistry, in situ hybridization, and immunofluorescence has revealed the presence of both SARS-CoV and SARS-CoV-2 in pancreatic tissues (including exocrine and endocrine cells) from individuals who died as a result of SARS and COVID-19.

SARS-CoV-2 has been shown to replicate in human pancreatic islets ex vivo, impairing *β*-cell functions and insulin secretion. It also affects reproductive tissue; the ACE2 receptor was expressed in testicular germ cells, Leydig cells, and Sertoli cells, as well as in ovarian, uterine, placental, vaginal, and breast tissues. SARS-CoV-2 was detected during testicular autopsy and in semen obtained from both COVID-19 patients and those recovering from COVID-19. Testicular damage and reduced testosterone levels were reported in men with COVID-19, as well as altered sex changes in women, as reported by a prospective Chinese study.

Infection with SARS-CoV-2 has been correlated with changes in menstrual flow, affecting 25% of women, and with a 19% increase in the duration of menstrual cycles. These changes are likely the result of temporary hormonal imbalances caused by the infection. Additionally, 36% of women with prolonged COVID-19 symptoms reported menstrual irregularities, including irregular cycles, unusually heavy bleeding, and instances of postmenopausal bleeding.

Certain treatments for SARS-CoV-2, such as glucocorticosteroids and low-molecular-weight heparin (LMWH), which are widely used, can affect the endocrine system. Treatment with glucocorticoids can cause hyperglycemia, insulin resistance, and dyslipidemia; prolonged use has been shown to inhibit the secretion of FSH, TSH, and LH, leading to adrenal suppression and overall adrenal insufficiency when the treatment is stopped. LMWH therapy used to prevent hypercoagulation associated with severe SARS-CoV-2 infections may interfere with the measurement of thyroid hormones without serum, which may result in a false increase [37]. Therefore, it is important to monitor patients with endocrine changes that occur during SARS-CoV-2 infection.

Thus, the endocrine system possesses the ACE2 enzyme and the TMPRSS2 protein, which promote the penetration of the SARS-CoV-2 virion into cells. In this regard, there is evidence that the endocrine system is susceptible to destruction and changes in function due to COVID-19.

#### 3.1.1. Pituitary Gland

ACE2 is poorly expressed in the pituitary gland [42]. Nevertheless, SARS-CoV mRNA was detected in the pituitary gland during autopsy [43], and a pathoanatomic study of five patients who died from SARS revealed a decrease in the number of somatotropic, thyroid-stimulating, and corticotropic cells and staining for GH, TSH, and ACTH [44]. In addition, the results of the study showed that ACTH is inhibited in critical patients with COVID-19, and SARS-CoV-2 may have a potential effect on adrenocorticotropic hormone through the ACE2 and AGTR1 proteins [42].

Several studies have reported that patients with Cushing's disease are at greater risk of contracting SARS-CoV-2 than the general population. Increased severity of COVID-19 was observed in patients with chronic uncontrolled hypercorticism [45, 46].

There are also cases of SARS-CoV-2 infection associated with pituitary apoplexy [47]. Thus, there may be an increased risk of PA in patients with pituitary tumors with COVID-19 infection, as indicated by several sources of disease cases. Although some of these sources indicated other risk factors for apoplexy, such as pregnancy and childbirth, preexisting pituitary macroadenomas were identified in most of them [47–49]. The specific involvement of COVID-19 in pathological conditions accompanied by hypopituitarism or hyponatremia is also known [46, 50]. In addition, patients with hypopituitarism may have concomitant diseases, such as diabetes mellitus, obesity, and spinal fractures, accompanied by a disturbance in carbohydrate metabolism and a general decrease in respiratory volume of the lungs, which, by themselves, both individually and collectively, may predispose them to severe COVID-19 [51, 52].

#### 3.1.2. Thyroid Gland

The effect of COVID-19 on thyroid function associated with preexisting thyroiditis and Graves' disease has been demonstrated [53, 54]. Cell culture studies revealed that the expression of TMPRSS2 and ACE2 in the thyroid gland was greater than that in the lungs [53, 55]. Consequently, the thyroid gland may be a potential target for SARS-CoV-2 [54, 55].

Cases of subacute thyroiditis caused by SARS-CoV-2 and postpartum thyroiditis are often detected in women in labor who have had a coronavirus infection during pregnancy [56, 57]. Studies have shown that COVID-19 can cause thyroid dysfunction in the acute phase and the recovery phase [54, 58]. Studies have shown that survivors of COVID-19 have a lower thyroid volume than control individuals, which is accompanied by a slight increase in TSH levels in this group of patients [59].

Morphologically, SARS-CoV-2 can disrupt the microstructure of the thyroid gland and cause damage to thyrocytes. These injuries can lead to a decrease in the volume of the thyroid gland for a long period, although the level of thyroid hormones fluctuates within small correlations [56, 60].

Isolated cases of Graves' disease after coronavirus infection have been noted [61].

Patients with autoimmune thyroid diseases may be more susceptible to the virus. Hypothyroidism is associated with increased mortality from coronavirus infections [62].

Graves' disease and Hashimoto's thyroiditis may occur a few months after subacute thyroiditis, which is considered a type of viral destructive thyroiditis [63].

The currently available information indicates that the function of the thyroid gland is restored with conservative treatment. In-depth and long-term studies are needed to improve our understanding and treatment of thyroid disease in patients with COVID-19.

#### 3.1.3. Pancreas

One study revealed that ACE2 and TMPRSS2 were expressed not only in respiratory tract organs but also in pancreatic islets [64–68]. Morphological and functional changes in islets, that is, a decrease in the number of insulin-secretory granules in *β*-cells, loss of insulin gene transcription, impaired insulin secretion, and a high number of bihormonal insulin/glucagon-positive cells, indicate the effect of SARS-CoV-2 on the pancreas [67, 69, 70].

Damage to pancreatic tissue can lead to a lack of control over blood glucose levels, which can result in the development of diabetes mellitus. Patients with reduced pancreatic function are at a greater risk of infection with COVID-19 [71].

*β*-cell dysfunction caused by infection can lead to an uncontrolled hyperglycemic state, especially in patients whose pancreas is already affected by diabetes mellitus [72]. In gestational diabetes mellitus, SARS-CoV-2 infection can lead to diabetic fetopathy [72].

The available data indicate that diabetes is the main risk factor for hospitalization and mortality [73, 74].

Many medicines used to treat diabetes (ACE inhibitors, angiotensin receptor blockers, ibuprofen, and thiazolidinediones) increase the secretion of ACE2 [75].

Data analysis has shown that patients with diabetes and concomitant CVDs, such as coronary heart disease, stroke, and peripheral artery disease, have an approximately 50% higher risk of mortality compared to those without CVDs [76]. This increased risk can be attributed to the compounded strain on the cardiovascular system and metabolic pathways, which are already compromised by both diabetes and COVID-19. Moreover, the presence of CVDs in diabetic patients often leads to more severe COVID-19 symptoms and complications, further elevating the likelihood of adverse outcomes.

There is also a hypothesis that SARS-CoV-2 causes hyperglycemia, but the literature on this topic is very scarce. Therefore, more in-depth studies on the effect of COVID-19 on the pancreas are needed.

#### 3.1.4. Adrenal Glands

The ACE2 receptor is present in the bundle and reticular zone of the adrenal cortex. However, TMPRSS2 is expressed in all three zones of the adrenal cortex [77]. Hypocorticism is observed in most patients who have had COVID-19 [78]. Autopsies of patients who died from COVID-19 showed hemorrhage in the adrenal glands, ischemic necrosis, and focal inflammation [77].

Clinically detected insufficiency of corticosteroids has also been reported in patients with the critical form of COVID-19 [79].

Histopathological examination of adrenal tissue sections revealed small vessel vasculitis (endothelins) in the periadrenal adipose tissue, increased perivascular lymphoplasmo-cellular infiltration of various densities, and, occasionally, mild extravasation of erythrocytes in the same zones and areas [80]. S proteins and SARS-CoV-2 mRNA were also detected directly in the cells of the adrenal cortex [80].

The consequences of coronavirus infection can include adrenal insufficiency and aggravate preexisting adrenal disease, including Addison's disease [81].

Chronic diseases, rheumatoid arthritis (RA), COPD, asthma, and prolonged use of glucocorticoids lead to adrenal insufficiency. Treatment with glucocorticoids also increases the mortality and severity of coronavirus infection [82].

Microscopic examination revealed acute fibrinoid necrosis of small vessels, mainly arterioles in the adrenal parenchyma and the adrenal capsule. Subendothelial vacuolization and apoptotic debris were also present [83].

Most of the published data indicate that the adrenal glands are a target for viral infection and, as a consequence, subsequent cell damage, which can contribute to adrenal dysfunction.

#### 3.1.5. Testes

Studies have shown the presence of SARS-CoV-2 in testicles, which indicates direct cytopathic damage to testicles with COVID-19 [84–86]. Histological studies of testicles from patients with COVID-19 revealed a significant decrease in germ cells, with germ cells almost completely absent in the vas deferens [84]. Patients with COVID-19 have testicular pain and epididymo-orchitis [87–89].

A decrease in sperm motility and normal morphology has been reported in men with COVID-19 [84, 90]. Studies conducted in China and Germany have shown high serum LH levels and low testosterone and FSH levels, which indicate direct and indirect cytotoxic damage to testicles [84, 91].

There are also data on the restoration of serum levels of LH, FSH, and testosterone in patients who recovered from COVID-19. Thus, the available data suggest that morphological changes occur in patients and can disrupt the function of even germ cells. However, there are data on the restoration of testosterone levels within a certain period after recovery [92].

There are studies indicating that high mortality rates from COVID-19 are associated with lower levels of total and free testosterone in men. This finding correlates with a similar effect on the male reproductive system of cigarette smoking, which also increases the expression of ACE2, which is why smokers also have a greater susceptibility to COVID-19 [93].

#### 3.1.6. Ovaries

There are very little data on the effect of COVID-19 infection on ovarian function. However, in international surveys of patients who underwent COVID-19, changes in the menstrual cycle and heavy bleeding were reported [94]. Thus, the effects of COVID-19 infection on the ovaries remain unclear and are likely to be mediated. Therefore, more in-depth studies on the effect of COVID-19 on the ovaries are needed.

### 3.2. Changes in the Functional Activity of Endocrine Organs During COVID-19

The SARS-CoV-2 virus has the ability to disrupt the function of several endocrine glands and metabolic processes, potentially leading to both acute and long-term endocrine or metabolic dysfunction. Patients with preexisting endocrine disorders or metabolic imbalances are at an increased risk of developing COVID-19, and such conditions may also worsen the severity of the disease, accelerating the progression toward critical complications and death. Additionally, SARS-CoV-2 binds to the ACE2 receptor and employs the cellular serine protease TMPRSS2 to activate the S protein, facilitating the virus's entry into host cells. This mechanism, combined with the virus's widespread effect on the endocrine system, underscores the complex interplay between COVID-19 and metabolic health.

The endocrine system contains ACE2 and TMPRSS2, which promote the penetration of SARS-CoV-2 virions into cells. In this regard, there is evidence that the endocrine system is vulnerable to both direct cytolytic action and indirect influences through changes in the functional activity of endocrine organs under the influence of COVID-19 on other organs and systems.

#### 3.2.1. Pituitary Gland

SARS-CoV-2 mRNA was detected in the pituitary gland during autopsy, and a pathoanatomic study of patients who died from SARS-CoV-2 revealed a decrease in the number of somatotropic, thyroid-stimulating, and corticotropic cells and in the levels of GH, TSH, and ACTH.

#### 3.2.2. Thyroid gland

Studies have shown that coronavirus can cause thyroiditis and Graves' disease, as well as a decrease in the volume of the thyroid gland, which is accompanied by a slight increase in TSH levels.

#### 3.2.3. Pancreas

Coronavirus infection leads to beta-cell dysfunction, which contributes to impaired insulin secretion. Damage to pancreatic tissue can lead to a lack of control over blood glucose levels, which can result in the development of diabetes mellitus. It can also result in uncontrolled hyperglycemia, especially in patients whose pancreas has already been affected by diabetes mellitus. The available data suggest that diabetes is the main risk factor for hospitalization and mortality.

In the adrenal glands, hypocorticism is observed in most patients who have had coronavirus. Autopsies of patients who died from COVID-19 showed hemorrhage in the adrenal glands, ischemic necrosis, and focal inflammation.

#### 3.2.4. Testicles

Histological studies of the testicles of patients with COVID-19 have shown a significant decrease in germ cells, with germ cells almost completely absent in the vas deferens. There is a high level of LH and low levels of testosterone and FSH.

## 4. Conclusion

An in-depth study and understanding of the impact of COVID-19 infection on the endocrine system will improve the rehabilitation of patients, as well as their health and quality of life. The endocrine system is more vulnerable to disorders caused by COVID-19 infection, mainly manifested by defects in thyroid function and hyperglycemia.

However, the aforementioned literature contains several unclear aspects and contradictory data, based on which much remains to be studied and clarified regarding the effects of COVID-19 on the endocrine system. In particular, this concerns the study of the hyperglycemic status of patients who have had coronavirus infection, which is extremely important for the future metabolic health of COVID-19 survivors. In addition, the study of changes in the morphological structure and functional activity of the gonads deserves special attention due to the lack of thorough studies and the importance of preserving and controlling the reproductive function and fertility of young patients who have undergone coronavirus infection. In particular, this requires the study of the state and functional activity of the ovaries in women.

The extent to which endocrine dysfunction affects the course of COVID-19 is currently unknown, so this area is the most important area for future research.
